# Accuracy of segment anything model for classification of vascular stenosis in digital subtraction angiography

**DOI:** 10.1186/s42155-025-00560-z

**Published:** 2025-05-19

**Authors:** Vagner Navasardyan, Maria Katz, Lukas Goertz, Vazgen Zohranyan, Hayk Navasardyan, Iram Shahzadi, Jan Robert Kröger, Jan Borggrefe

**Affiliations:** 1 Department of Radiology, Neuroradiology and Nuclear Medicine, Johannes Wesling University Hospital, Hans-Nolte-Straße 1, 32429 Minden, Germany; 2 Ruhr University Bochum, Universitätsstraße 150, 44801 Bochum, Germany; 3 Department of Diagnostic and Interventional Radiology, Uniklinik Köln, Kerpener Str. 62, 50937 Cologne, Germany; 4 ServiceTitan, Inc, 11/1 Antarain St, 0009 Yerevan, Armenia; 5 Synopsys Armenia CJSC, 41 Arshakunyats Ave, Yerevan, Armenia

**Keywords:** Artificial intelligence (AI), Segment Anything Model (SAM), Vascular segmentation; peripheral arterial disease, Digital subtraction angiography

## Abstract

**Background:**

This retrospective study evaluates the diagnostic performance of an optimized comprehensive multi-stage framework based on the Segment Anything Model (SAM), which we named Dr-SAM, for detecting and grading vascular stenosis in the abdominal aorta and iliac arteries using digital subtraction angiography (DSA).

**Materials and methods:**

A total of 100 DSA examinations were conducted on 100 patients. The infrarenal abdominal aorta (AAI), common iliac arteries (CIA), and external iliac arteries (EIA) were independently evaluated by two experienced radiologists using a standardized 5-point grading scale. Dr-SAM analyzed the same DSA images, and its assessments were compared with the average stenosis grading provided by the radiologists. Diagnostic accuracy was evaluated using Cohen's kappa, specificity, sensitivity, and Wilcoxon signed-rank tests.

**Results:**

Interobserver agreement between radiologists, which established the reference standard, was strong (Cohen's kappa: CIA right = 0.95, CIA left = 0.94, EIA right = 0.98, EIA left = 0.98, AAI = 0.79). Dr-SAM showed high agreement with radiologist consensus for CIA (κ = 0.93 right, 0.91 left), moderate agreement for EIA (κ = 0.79 right, 0.76 left), and fair agreement for AAI (κ = 0.70). Dr-SAM demonstrated excellent specificity (up to 1.0) and robust sensitivity (0.67–0.83). Wilcoxon tests revealed no significant differences between Dr-SAM and radiologist grading (*p* > 0.05).

**Conclusion:**

Dr-SAM proved to be an accurate and efficient tool for vascular assessment, with the potential to streamline diagnostic workflows and reduce variability in stenosis grading. Its ability to deliver rapid and consistent evaluations may contribute to earlier detection of disease and the optimization of treatment strategies. Further studies are needed to confirm these findings in prospective settings and to enhance its capabilities, particularly in the detection of occlusions.

## Introduction

Narrowing of blood vessels due to atherosclerotic plaque buildup is a critical factor in the progression of peripheral arterial disease (PAD) [1], affecting over 235 million people globally. In Germany, PAD affects 6–8% of the population (8.2% of men and 5.5% of women), reflecting its status as a manifestation of systemic atherosclerosis [2]. In the U.S., the prevalence of PAD increased from 11.3 million in 1995 to 21.0 million by 2020, indicating a significant rise in this condition [3]. Early detection and precise grading of stenosis are crucial for determining the appropriate treatment strategies [4]. Accurate assessment of stenosis is essential for improving patient outcomes in these populations [5, 6].

Traditional imaging techniques, such as Digital Subtraction Angiography (DSA), Magnetic Resonance Angiography (MRA) and Computed Tomography Angiography (CTA), are considered the reference standard for vascular assessment [7, 8]. While CTA and MRA are frequently used for initial diagnosis, DSA continues to play a key role in guiding therapeutic interventions due to its precision in procedural decision-making [9]. However, these techniques are resource-intensive and require significant expertise, along with manual interpretation by radiologists. This manual process leads to inter-reader variability and increases the workload for radiologists, potentially affecting the consistency and efficiency of diagnostic assessments. The use of automated tools can improve diagnostic efficiency and consistency in imaging analysis [10].

Several studies have demonstrated the effectiveness and developmental potential of AI in evaluating radiological examinations [11, 12]. For example, in neuroradiology, AI has been applied in assessing intracranial aneurysms [13], automated detection of arterial landmarks and vascular occlusions in patients with acute stroke undergoing DSA with deep learning [14], and assessment of vascular involvement and tumor resectability on computed tomography [15]. The choice of SAM over other AI models was driven by its zero-shot learning capability, flexibility, and the ability to handle complex segmentation tasks in angiography images with minimal setup or fine-tuning. These features make SAM an ideal candidate for tackling the challenges of vascular segmentation and anomaly detection in peripheral angiographic images.

In this study, we utilize an optimized advanced multistage framework, which we named Dr-SAM [16], built on the AI-driven Segment Anything Model (SAM) [17]. Dr-SAM is specifically designed to detect vascular anomalies such as narrowing (stenosis) and dilation (aneurysms) in angiography. This retrospective study evaluates Dr-SAM’s accuracy in detecting and grading stenosis in the abdominal aorta and iliac arteries using DSA, comparing its results with radiologists’ assessments to determine reliability and potential workflow improvements. We hypothesize Dr-SAM will show strong agreement with manual DSA evaluations.

## Materials and methods

### Patients

This retrospective double-center study was approved by the local ethics committee (2024–1199). Due to the retrospective nature of the study, the need for informed consent was waived.

The study included 100 adult patients who underwent DSA of the abdominal and pelvic arteries between May 2019 and January 2024 at two large vascular centers. Patients were selected based on predefined inclusion criteria: age ≥ 18 years and having undergone diagnostic or therapeutic DSA of the infrarenal abdominal aorta and iliac arteries. Patients were excluded if they had a history of endovascular aneurysm repair (EVAR), open aortic repair (OAR) with Y or tube prosthesis, unilateral aorto-iliac or aortofemoral bypass, known occlusion of the abdominal aorta, bilateral pelvic artery occlusions, or previous spinal surgery involving metal implants. Initially, 138 patients were screened; 26 were excluded due to prior EVAR/OAR (*n* = 11), unilateral aorto-iliac or aortofemoral bypasses (*n* = 5), bilateral pelvic artery occlusions (*n* = 7), known occlusion of the abdominal aorta (*n* = 4), and previous spinal surgery with metal implants (*n* = 9). Thus, 100 consecutive eligible patients were included for final analysis.

#### DSA

**Fig. 1:**
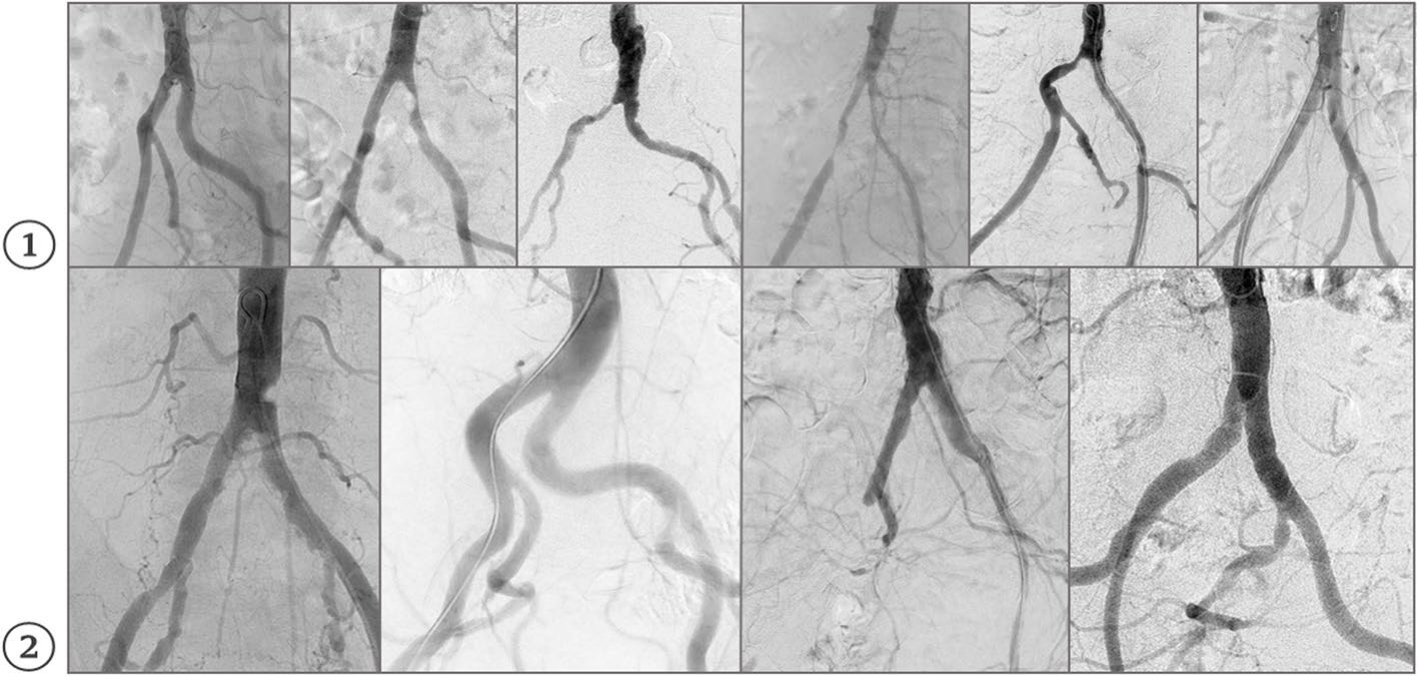
DSA image example: cropped and anonymized. 1. 386 × 448 pixels, 2. 818 × 946

DSA examinations were conducted using the Allura Xper FD20/15 (Philips Healthcare, Netherlands) and Artis icono floor (Siemens Healthineers AG, Germany) angiography systems. A 20 mL dose of iodinated contrast agent was administered at 10 mL/sec via a 4F UF or Pigtail catheter using an Accutron HP-D injector (Medtron). Additive images of the infrarenal abdominal aorta and pelvic arteries were anonymized, saved in JPEG format, and standardized using Adobe Photoshop 2024. Images were cropped to 818 × 946 pixels (Artis icono floor) or 386 × 448 pixels (Allura Xper FD20/15) (Fig. 1) [18, 19].

Two board-certified radiologists (15 and 12 years of experience) independently assessed the infrarenal abdominal aorta (AAI), right and left common iliac arteries (CIA), and right and left external iliac arteries (EIA) for stenosis severity. Evaluations were performed on additive (processed) images. Stenosis was graded from 1 to 5 based on severity (Table 1). Results were documented per vascular segment (Table 2).

**Table 1:** 5-point grading scale

Grade	Description	Stenosis range (%)
1	No stenosis or non-relevant stenosis	0—24%
2	Mild stenosis	25—50%
3	Moderate stenosis	51—75%
4	Severe stenosis	76—99%
5	Occlusion	100%

**Table 2:** Summary of DSA examination protocols, image processing, and evaluation criteria

**Aspect**	**Details**	**Number**
Angiography systems	Allura Xper FD20/15 (Philips Healthcare, Netherlands)	51
	Artis icono floor (Siemens Healthineers AG, Germany)	49
Contrast agent administration	Dose: 20 mL	100
	Flow Rate: 10 mL/sec	100
	Catheter Types: 4F UF / 4F Pigtail	69/31
	Injector: Accutron HP-D high-pressure injector (Medtron)	100
Imaging area	infrarenal abdominal aorta and pelvic arteries	100
Evaluated vascular segments	Infrarenal abdominal aorta (AAI)	100
	Right and Left Common Iliac Arteries (CIA right, CIA left)	100
	Right and Left External Iliac Arteries (EIA right, EIA left)	100
Evaluators	Two board-certified radiologists with 15 and 12 years of experience in vascular imaging	
Image processing and format	Software: Adobe Photoshop	
	Resolution Settings	
	Artis icono floor: 818 x 946 pixels	49
	Allura Xper FD20/15: 386 x 448 pixels	51
	JPEG (anonymized), cropped	100

### Our framework

Dr-SAM based on Segment Anything Model (SAM) [17] is a universal algorithm for anomaly detection in blood vessels, incorporating zero-shot technology for vessel extraction followed by anomaly detection with the integration of topological skeleton.

Using Meta AI's SAM model for segmentation, we improve vessel extraction from X-ray images by selecting precise positive points, as SAM allows using positive and negative points as prompts to refine segmentation. Initial selection involves generating a probability map, filtering low-probability pixels, and sampling 100 points to avoid clustering, with the densest point chosen as positive. A second point is selected similarly. This process repeats three times, predicting with previously found points and identifying the next point outside the predicted mask, yielding five positive points for the final segmentation [16].

**Fig. 2:**
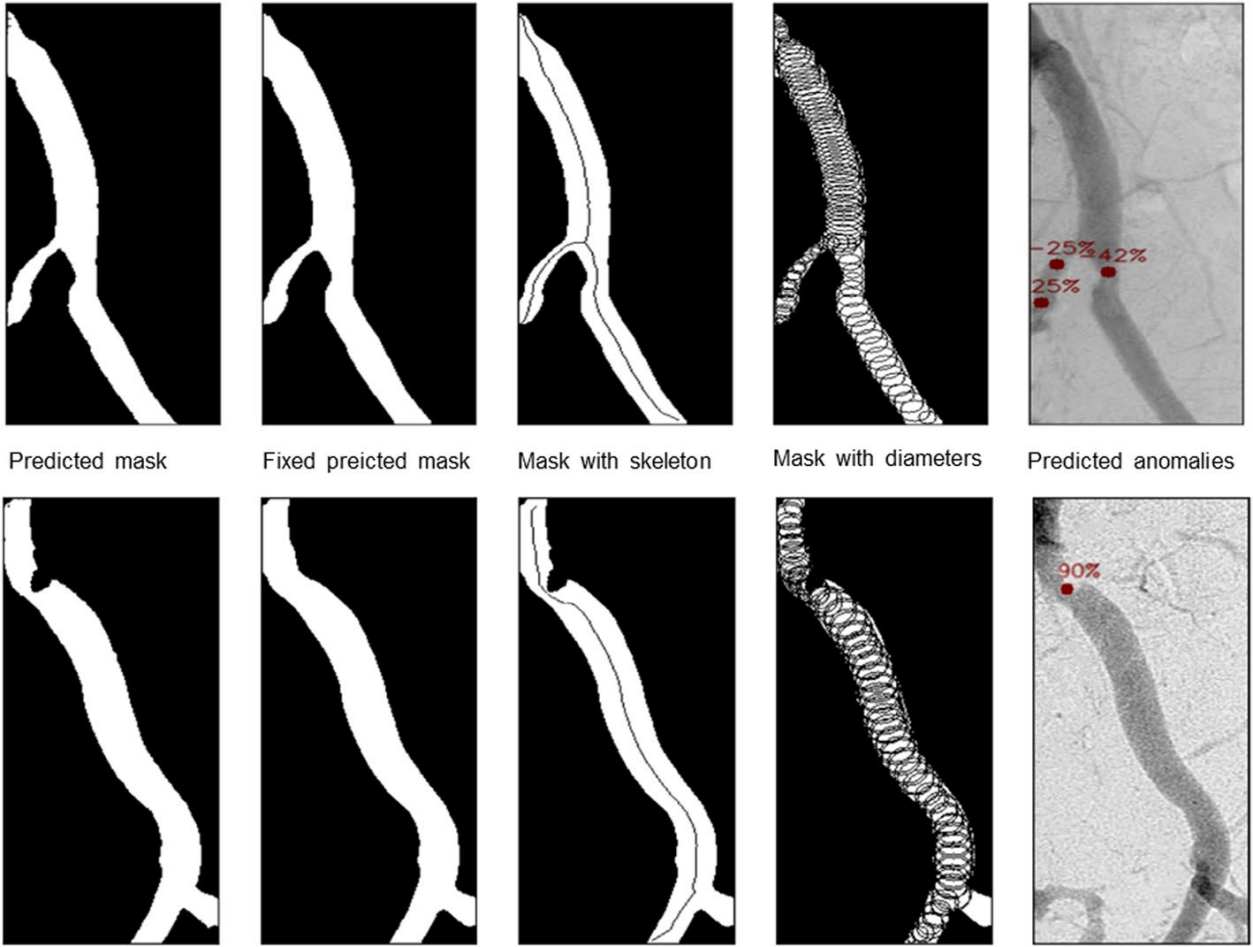
Our framework pipeline results for each step

For anomaly detection, we utilized a topological skeleton [20], a method widely employed in X-ray image studies within Computer Vision. The topological skeleton is a vital component in identifying or approximating the centerline of a vessel, aiding in the determination of its diameter in specific regions. Our proposed algorithm involves the use of a topological skeleton, which is subsequently pruned by removing unnecessary branches while preserving the vessel’s structural integrity (Fig. 2). To do so, our algorithm begins by extracting branches from the vessel’s topological skeleton. Once the branches are separated, we identify and remove those that are below a certain length threshold, as these short branches are likely artifacts resulting from anomalies in the vessel structure rather than true branches. Next, we analyzed the extracted skeleton segments by approximating diameters along each segment to identify anomalies. We focused on the highest and lowest diameters within each segment, assuming these extremes indicate potential anomalies. In the final step, we applied a threshold to the percentile change between these extremum points and their surrounding points, enabling more accurate anomaly detection. After detecting anomaly points we leverage the distance transform technique [21] to better estimate the diameter near the anomaly points. Calculating the percentile change of the distance transform function values between anomaly points and their surrounding points, we eventually got the estimate of the percentage of the anomaly. Currently, Dr-SAM is unable to detect occlusions in blood vessels, as these areas lack a skeleton representation, preventing the algorithm from accounting for those regions.

### Training and validation process

Dr-SAM is based on the pre-trained Segment Anything Model (SAM) and uses its zero-shot learning capabilities for vessel segmentation without the need for additional fine-tuning. To evaluate the performance of our positive point selection strategy and the anomaly detection method, we tested Dr-SAM on a benchmark dataset of 500 peripheral angiographic images. This dataset, introduced in our previous publication [16], contains expert-labeled vessel masks and annotated anomalies. We assessed Dr-SAM’s performance by comparing its results to these ground truth labels, using Intersection over Union (IoU) for segmentation quality and precision-recall metrics for anomaly detection. The validation results confirmed that Dr-SAM performs reliably across different angiographic datasets, highlighting its robustness and potential for clinical application.

The details of both the segmentation and anomaly detection algorithms, including point selection, threshold definition, and other aspects, have been described in our last paper [16].

### Statistical analysis

The average stenosis grade from two observers in DSA served as the “reference standard”. Stenosis degrees across vascular segments were compared using the Wilcoxon matched-pairs test, with p-values < 0.05 considered statistically significant. Interobserver and intermeshed agreement was evaluated with Cohen’s weighted kappa coefficients (κ) and Spearman rank correlation coefficients (r). Kappa values were interpreted as follows: 0.4 or less (poor), 0.41–0.6 (moderate), 0.61–0.8 (good), and 0.81–1.0 (excellent agreement). The strength of the correlation coefficient is interpreted as: 0.1 <|r|≤ 0.3 is considered a weak correlation, 0.3 <|r|≤ 0.5 is considered a moderate correlation |r|> 0.5 is considered a strong correlation. Sensitivity, specificity, positive predictive value (PPV) and negative predictive value (NPV) were calculated for OSMF and DSA methods in high-grade (4,5) and moderate categories (1,2,3) of stenosis in each vascular segment. Statistical analysis was conducted using Python version 3.9 with scipy version 1.10.1 and statsmodel 0.14.0 packages.

## Result

The high inter-rater agreement between the two radiologists in DSA assessments (κ: CIA right = 0.95, CIA left = 0.94, EIA right = 0.97, EIA left = 0.98, AAI = 0.79) allowed the use of their average readings as the final DSA value, which served as the reference standard.

Among the 500 vessels evaluated using DSA 20 (4%) were identified as occluded, 32 (6,4%) demonstrated severe stenosis (76%–99%), 24 (4.8%) showed moderate stenosis (50%–75%), 44 (8,8%) exhibited mild stenosis (< 50%), 380 (76.0%) were classified as having no stenosis.

**Fig. 3:**
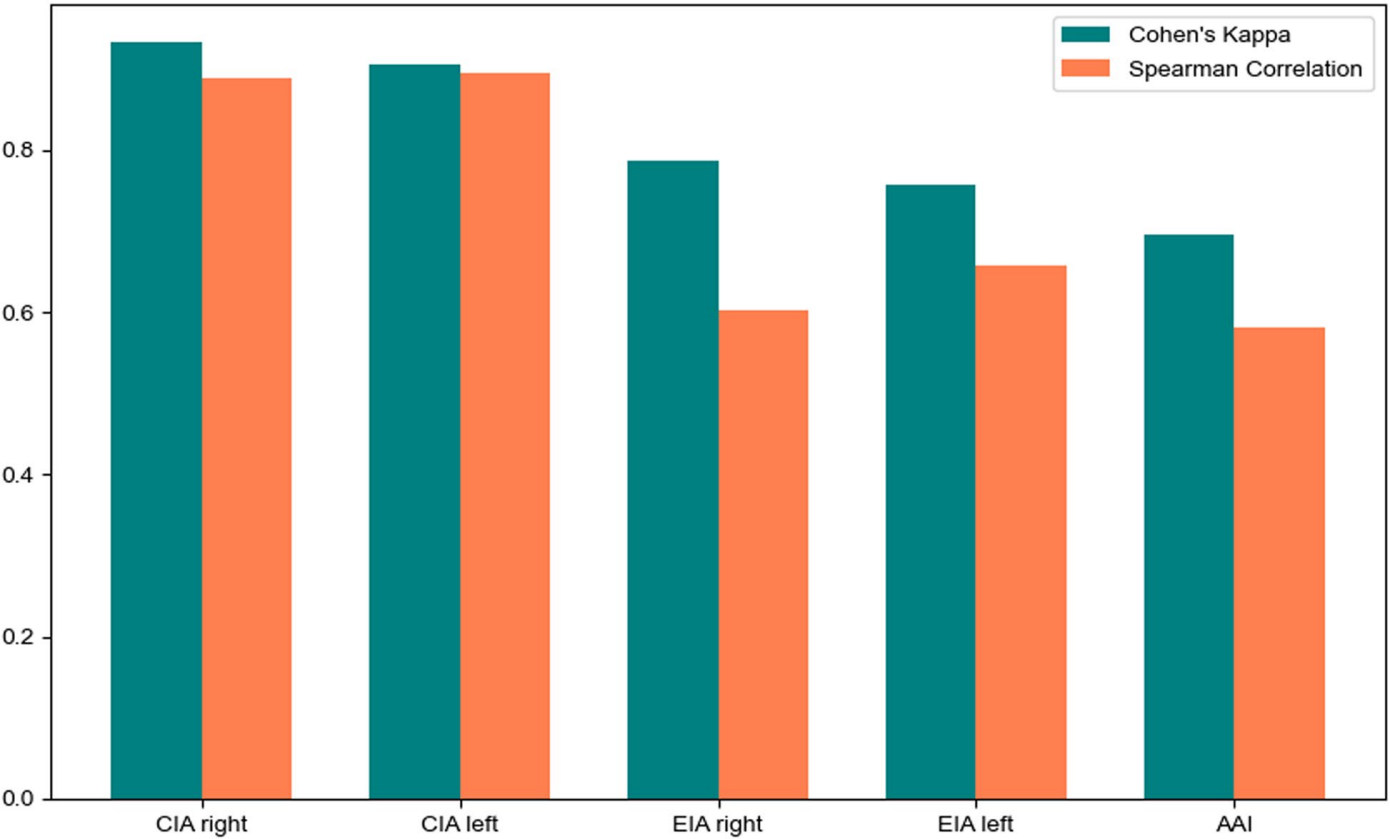
Cohen’s weighted kappa scores and Spearman Correlation analysis between DSA vs Dr-SAM (severe stenosis)

The degree of stenosis on Dr-SAM was highly correlated with DSA for CIA right (κ = 0.93, r = 0.89, *p* > 0.05) and CIA left (κ = 0.91, r = 0.90, *p* > 0.05). A slightly lower correlation was observed for EIA right (κ = 0.79, r = 0.60, *p* > 0.05) and EIA left (κ = 0.76, r = 0.66, *p* > 0.05). In general, Dr-SAM tended to underestimate mild and moderate stenosis. For the EIA right, mild stenosis was reported as 68% by Dr-SAM compared to 76% by DSA, and moderate stenosis as 4% by Dr-SAM compared to 9% by DSA. For the EIA left, Dr-SAM underestimated mild stenosis (69% vs. 76% by DSA), while moderate stenosis was identical between methods (5% vs. 5%). The lowest, but still acceptable, agreement was found for the AAI (κ = 0.70, r = 0.58, *p* > 0.05), where Dr-SAM underestimated mild stenosis (82% vs. 87% by DSA) and moderate stenosis (5% vs. 11% by DSA). This underestimation tendency mainly affected lower-grade stenoses (mild and moderate), while severe stenoses were reliably detected. Importantly, no relevant underestimation occurred for severe stenoses across all evaluated vessels, which is crucial for clinical decision-making, where identifying higher-grade stenosis has the greatest therapeutic relevance (Fig. 3).

**Fig. 4:**
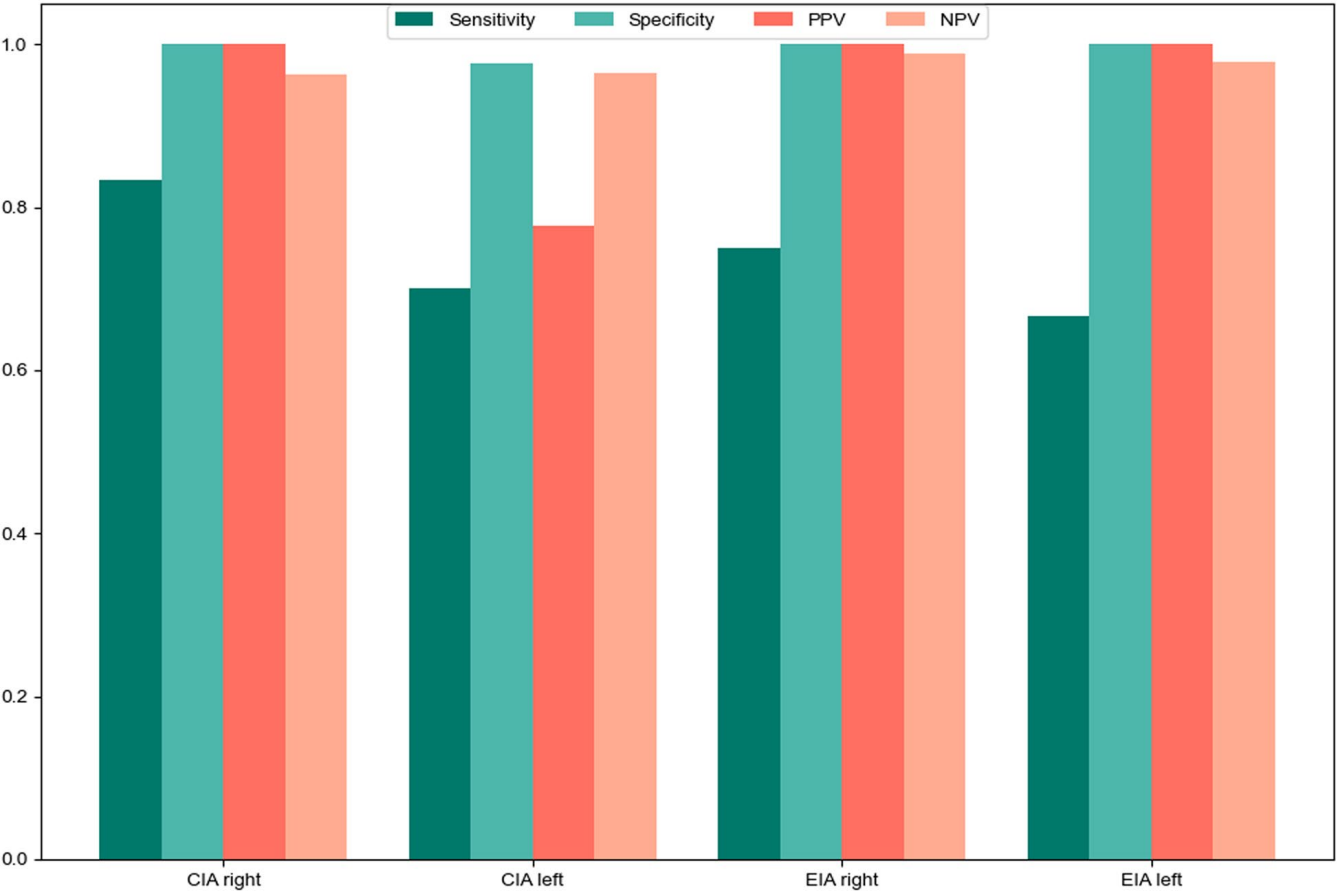
Sensitivity and Specificity, PPV, NPV analysis (DSA as ground truth)

Using radiologists’ findings as the reference standard, the diagnostic performance of Dr-SAM was evaluated based on sensitivity, specificity, positive predictive value (PPV), and negative predictive value (NPV) (Fig. 4). For the CIA right, Dr-SAM demonstrated a sensitivity of 0.83, specificity of 1.00, PPV of 1.00, and NPV of 0.96. Similarly, the CIA left exhibited a sensitivity of 0.70, specificity of 0.98, PPV of 0.78, and NPV of 0.97. In the EIA regions, the EIA right showed a sensitivity of 0.75, specificity of 1.00, PPV of 1.00, and NPV of 0.99, while the EIA left had a sensitivity of 0.67, specificity of 1.00, PPV of 1.00, and NPV of 0.98. For the infrarenal AAI, all evaluations on DSA were classified as low-grade stenosis or no, and Dr-SAM successfully identified these cases without discrepancies.

Dr-SAM exhibited consistently high specificity, reaching 100% in most regions, alongside good sensitivity across all vessels. The diagnostic performance was particularly robust in detecting stenosis within the CIA and EIA regions. These findings highlight Dr-SAM’s potential as a reliable tool for vascular assessments across various anatomical regions.

## Discussion

This study highlights the potential of the Dr-SAM as an automated tool for vascular image analysis, particularly in detecting and grading stenosis in the pelvic vessels imaged with DSA. The results demonstrate strong concordance between Dr-SAM and manual DSA evaluations, with Cohen’s kappa indicating excellent agreement for the CIA (κ = 0.93–0.91), moderate agreement for the EIA (κ = 0.79–0.76), and fair agreement for the AAI (κ = 0.70). Dr-SAM achieved excellent specificity (up to 1.0) and robust sensitivity (0.67–0.83), making it a reliable tool for stenosis detection, despite some limitations.

The study underscores Dr-SAM’s ability to deliver consistent stenosis measurements, addressing a critical challenge in clinical practice: variability in human interpretation. Its excellent agreement in the CIA and moderate agreement in the EIA regions reflect its capacity to accurately assess vascular stenosis across different observers and modalities. However, Dr-SAM tended to underestimate mild and moderate stenosis in certain regions. For example, in the EIA, mild stenosis was reported as 68–69% by Dr-SAM compared to 76% by DSA, and moderate stenosis as 4–5% versus 9–11%. Importantly, Dr-SAM showed no underestimation of severe stenosis, which is crucial for clinical decisions.

A significant limitation of this study is Dr-SAM’s current inability to detect occlusions, due to the lack of skeletal representation in completely blocked vessels. These cases were excluded from analysis, potentially lowering overall concordance with DSA. Clinically, the inability to detect occlusions can result in incomplete diagnostic assessments, potentially delaying appropriate treatment decisions such as bypass surgery or thrombolysis. Future iterations of Dr-SAM should incorporate advanced AI models capable of flow analysis, collateral mapping, and vessel reconstruction to address this limitation and improve clinical outcomes. Potential biases inherent to retrospective studies must also be considered, including selection bias and the use of previously collected data, which may not fully represent current clinical practices or adequately capture patient variability.

Dr-SAM has the potential to optimize vascular imaging workflows by automating stenosis grading. Its high specificity and sensitivity make it a valuable tool for planning interventions such as angioplasty or stent placement. The precise performance of Dr-SAM in complex regions such as the abdominal aorta and iliac arteries underscores its potential applicability to other vascular territories. By reducing variability in assessments and accelerating diagnostic processes, Dr-SAM could improve decision-making and patient outcomes.

Future research should include prospective validation studies with larger, more diverse patient populations to confirm Dr-SAM’s effectiveness in real-world clinical scenarios. In addition, comparative studies should be conducted, although there are currently very few similar publications available. Most existing studies focus primarily on AI-based vessel segmentation, such as [22] and [23]. One of the few publications specifically addressing AI-based automated stenosis detection of vessels also demonstrated promising results [24]. However, a direct comparison is challenging because the study examines vessels from different anatomical regions and applies different imaging and analysis methods. These points highlight the need for further research in AI-based stenosis detection.

One of Dr-SAM's significant advantages is its potential to improve workflow efficiency in vascular imaging. Manual evaluation of vascular images is often time-consuming and requires expertise. Dr-SAM's integration into clinical practice could help automate routine tasks, enabling radiologists to focus on more complex cases or patient management. However, introducing AI tools in clinical settings requires proper training for radiologists to interpret AI-generated results and clear awareness of its limitations, particularly Dr-SAM's inability to detect occlusions, to ensure appropriate clinical interpretation and management.

Increasingly, publications [10, 25–33] highlight AI's growing role in radiology, demonstrating potential improvements in diagnostic accuracy, standardization of assessments, and efficiency enhancements across various imaging modalities. However, a direct comparison of Dr-SAM with other similar AI-based vascular imaging tools is still lacking, and addressing this gap should be a priority for future studies.

## Conclusion

In conclusion, the Dr-SAM is a promising tool for automated vascular stenosis assessment, and shows high concordance with manual DSA evaluations. While its current limitation in detecting occlusions requires further refinement, its accuracy and consistency support its potential to streamline workflows and enhance diagnostic efficiency. Future research should focus on validating these findings in real-time clinical settings, applying Dr-SAM to other vascular regions, and improving occlusion detection capabilities. With continued development, Dr-SAM could play a vital role in managing vascular conditions, particularly peripheral arterial disease.

## Data Availability

The Dr-SAM framework is publicly available at https://github.com/vazgenzohranyan/Dr.SAM. Data from the retrospective study analyzed in this article are available from the corresponding author upon reasonable request, subject to institutional and ethical regulations.

## Abbreviations

AAI: Infrarenal Abdominal Aorta
AI: Artificial Intelligence
APC: Article Processing Charge
CIA: Common Iliac Artery
CTA: Computed Tomography Angiography
DFG: Deutsche Forschungsgemeinschaft (German Research Foundation)
DSA: Digital Subtraction Angiography
EIA: External Iliac Artery
EVAR: Endovascular Aneurysm Repair
JWK: Johannes Wesling Klinikum
NPV: Negative Predictive Value
OAK: Open Access Publication Costs
OAR: Open Aortic Repair
PAD: Peripheral Arterial Disease
PPV: Positive Predictive Value
RUB: Ruhr University Bochum
SAM: Segment Anything Model
